# Genetic Interactions Between Brassinosteroid-Inactivating P450s and Photomorphogenic Photoreceptors in *Arabidopsis thaliana*

**DOI:** 10.1534/g3.112.004580

**Published:** 2012-12-01

**Authors:** Kulbir Singh Sandhu, Katherine Hagely, Michael M. Neff

**Affiliations:** * Department of Crop and Soil Sciences, Washington State University, Pullman, Washington 99164; † College of Agriculture, Food and Natural Resources, University of Missouri, Columbia, Missouri 65211

**Keywords:** CYP734A1, CYP72C1, phytochrome A, phytochrome B, cryptochrome 1

## Abstract

Plants use light as a source of information via a suite of photomorphogenic photoreceptors to optimize growth in response to their light environment. Growth-promoting hormones such as brassinosteroids also can modulate many of these responses. BAS1 and SOB7 are brassinosteroid-catabolizing P450s in *Arabidopsis thaliana* that synergistically/redundantly modulate photomorphogenic traits such as flowering time. The role of BAS1 and SOB7 in photomorphogenesis has been investigated by studying null-mutant genetic interactions with the photoreceptors phyA, phyB, and cry1 with regard to seed germination and flowering time. The removal of *BAS1* and/or *SOB7* rescued the low germination rate of the *phyA-211 phyB-9* double-null mutant. With regard to floral induction, *bas1-2* and *sob7-1* showed a complex set of genetic interactions with photoreceptor-null mutants. Histochemical analysis of transgenic plants harboring *BAS1*:BAS1-GUS and *SOB7*:SOB7-GUS translational fusions under the control of their endogenous promoters revealed overlapping and distinct expression patterns. BAS1’s expression in the shoot apex increases during the phase transition from short-to-long-day growth conditions and requires phyB in red light. In summary, *BAS1* and *SOB7* displayed both simple and complex genetic interactions with the phytochromes in a plant-stage specific manner.

In angiosperm plant species, the timing of flowering plays an important role in the success of sexual reproduction. Precision in the timing of reproduction requires a flowering mechanism that is both flexible and robust. Flexibility ensures that the timing of flowering leads to good seed set and survival under a variety of circumstances. Robustness ensures that the mechanism is strong enough to trigger flowering in the majority of members in a population. Cues that regulate floral induction include both environmental (external) and developmental (internal) factors. Being sessile and photosynthetic, light is a major environmental factor for plants. Light-mediated development, or photomorphogenesis, plays an important role in the optimization of flowering time (Mockler *et al.* 2003). Internal factors that affect flowering time include hormones such as brassinosteroids (BRs), as well as developmental factors, which include age. In plants, these external and internal cues are perceived by various reproductive pathways, which ultimately converge to ensure a proper flowering response (Srikanth and Schmid 2011). One of the fundamental questions in plant biology relates to how plants integrate light and hormone signals to optimize growth and development in constantly changing environmental conditions.

Plants perceive light quality and quantity via a suite of signal-transducing photoreceptors to facilitate adaption to their ambient environment. In *Arabidopsis* there are five red/far-red–absorbing phytochromes (phyA through phyE), two blue light–absorbing cryptochromes, (cry1 and cry2), and two blue/ultraviolet light–absorbing phototropins (phot1 and phot2) (Chory 2010). phyA is the most important far-red light sensor in *Arabidopsis*. A role of phyA as the major far-red sensor is supported by the observation that far-red light–grown *PHYA* loss-of-function mutants phenocopy dark-grown wild-type plants (Nagatani *et al.* 1993; Parks and Quail 1993; Whitelam *et al.* 1993; Neff and Chory 1998). phyB is a major regulator of seedling deetiolation in response to both white and red light, with null alleles conferring elongated hypocotyls in both conditions (Somers *et al.* 1991; Reed *et al.* 1993, 1994). cry1 and cry2 regulate deetiolation in response to medium- and low-intensity blue light, respectively (Ahmad and Cashmore 1993; Lin *et al.* 1998). In addition to seedling de-etiolation, some of these photoreceptors also mediate floral induction in responses to changing light conditions.

*Arabidopsis* is a facultative long-day plant and employs photoperiodic flowering pathways to accelerate flowering under long-day conditions. phyA plays a vital role in the photoperiodic flowering pathway by perceiving changes in day length. *PHYA*-null mutant plants are insensitive to floral induction by day-length extensions or night-break light treatments for short-day–grown plants, both of which mimic long-day growth conditions (Johnson *et al.* 1994; Reed *et al.* 1994). In addition, under long-day growth conditions, *PHYA*-null mutant plants display a late-flowering phenotype when compared with the wild type (Johnson *et al.* 1994; Neff and Chory 1998). phyB, on the other hand, inhibits flowering in *Arabidopsis*. Loss of phyB accelerates flowering under both long- and short-day conditions (Goto *et al.* 1991; Whitelam and Smith 1991; Halliday *et al.* 1994).

In addition to the flowering phenotype, *phyB* mutants are severely pleiotropic, demonstrating their widespread importance in *Arabidopsis* development (for review, see Franklin and Quail 2010). To identify downstream components of phyB signaling, various loss-of-function genetic approaches have been used, including the identification of mutants that either mimic or suppress *phyB*-null phenotypes (Reed *et al.* 1998, 2000). To complement these loss-of-function approaches, we used an activation-tagging screen to identify gain-of-function downstream components that may act in a redundant manner (Weigel *et al.* 2000). *PHYB-4 ACTIVATION-TAGGED SUPPRESSOR #1-DOMINANT* (*bas1-D*) and *SUPPRESSOR OF PHYB-4 #7-DOMINANT* (*sob7-D*) were both identified in a gain-of-function activation-tagging screen for suppressors of the photomorphogenic phenotypes conferred by a weak mutation of *PHYB* (Neff *et al.* 1999; Turk *et al.* 2005). BAS1 and SOB7 are members of the cytochrome P450 mono-oxygenase superfamily (P450s). Members of the P450 superfamily catalyze oxidation of a diverse array of plant metabolites. Reactions catalyzed by P450s are highly substrate specific to an extent that even close P450 family members may have widely diverse biochemistries as well as substrate requirements (for review see, Schuler *et al.* 2006).

Overexpression of either *BAS1/CYP734A1* or *SOB7/CYP72C1* suppresses the long-hypocotyl phenotype of *phyB-4* and also confers a BR-deficient phenotype typified by *DE-ETIOLATED 2-1* (*det2-1*), a BR biosynthetic mutant. Molecular, biochemical, and genetic analyses have shown that despite the high sequence similarity at both DNA and protein levels, BAS1 and SOB7 each catabolize their own specific substrates with unique biochemistries. BAS1 hydroxylates brassinolide, the most active BR in *Arabidopsis* and its immediate precursor castasterone to their respective inactive C-26 hydroxy products (Turk *et al.* 2005). SOB7, on the other hand, is not a C-26 hydroxylase and seems to act on precursors of BR biosynthesis (Turk *et al.* 2003, 2005; Thornton *et al.* 2010). BR levels are elevated in the *bas1-2 sob7-1* double-null mutant when compared with the wild-type or either single-null allele (Turk *et al.* 2005). *BAS1* and *SOB7* also affect developmental processes, such as flowering, in a synergistic/redundant fashion. In a manner quantitatively similar to *phyB*-null mutants, the *bas1-2 sob7-1* double-null flowers earlier than the wild-type in both long- and short-day growth conditions, demonstrating a role for BR inactivation in floral induction (Turk *et al.* 2005).

BRs are growth-promoting hormones essential for normal development of plants (Sasse 2003). BRs affect plant growth and development by altering the expression of hundreds of BR responsive genes (Goda *et al.* 2002). In addition to their general role in cell division and expansion (Bajguz 2000), BRs are also involved in tissue-specific development (Yamamoto *et al.* 1997; Symons *et al.* 2006). Unlike most plant hormones, however, BRs are not transported within or between plant tissues, implying that levels of BRs are regulated locally through both biosynthesis and catabolism (Reid and Ross 1989; Symons and Reid 2004; Montoya *et al.* 2005; Savaldi-Goldstein *et al.* 2007). BR catabolism, therefore, can play a significant role as a regulatory point for BR-mediated development. In fact, apart from BAS1 and SOB7, there are at least five more enzymes with unique biochemistries leading to BR inactivation in *Arabidopsis* (Poppenberger *et al.* 2005; Marsolais *et al.* 2007; Yuan *et al.* 2007; Husar *et al.* 2011). Independent evolution of multiple BR inactivating pathways further indicates the importance of this process in plant growth and development. Therefore, identifying the contributions of enzymes and pathways related to the inactivation of these hormones is important for understanding BR-mediated development.

The observation that photomorphogenic photoreceptors, along with and *BAS1* and *SOB7*, affect common developmental processes suggests that in at least some cases these pathways act in an interdependent manner. In the present work, we describe the contribution of BAS1 and SOB7 in the modulation of seed germination and flowering time in *Arabidopsis*. Genetic interactions for seed germination and flowering time were studied between *BAS1*, *SOB7* and the photoreceptors *PHYA*, *PHYB* and *CRY1* using null-mutant combinations. Our results indicate that both *BAS1* and *SOB7* contribute to the rate of seed germination in a manner that is genetically independent and/or downstream of *PHYA* and *PHYB*. *BAS1* and *SOB7* also show complex genetic interactions with *PHYA* and *PHYB* for flowering time. For example, *bas1-2* and *sob7-1* single-nulls have a mutually distinct pattern of genetic interactions with a *phyA* null. In contrast, the early-flowering phenotype of the *bas1-2 sob7-1* double null requires functional phyB. Furthermore, we show that BAS1 and SOB7 have both unique and overlapping expression patterns in *Arabidopsis*, and that BAS1 expression in the shoot apex in red light is dependent on the presence of functional phyB.

## Materials and Methods

### Plant material

All mutants used in this study, *phyA-211* (Reed *et al.* 1994), *phyB-9* (Reed *et al.* 1993), *cry1-103* (Liscum and Hangarter 1991), *bas1-2*, and *sob7-1* (Turk *et al.* 2005), were in the Columbia (Col-0) background. The *phyA-211* mutation was identified by amplifying genomic DNA with primers 5′-GTC ACA AGA TCT GAT CAT GGC-3′, 5′-AAC AAC CGA AGG GCT GAA TC-3′, 5′-TTA TCC ACA GGG TTA CAG GG-3′, and 5′-GCA TTC TCC TTG CAT CAT CC-3′, followed by resolution of 1243- and 1136-bp fragments for the wild-type and a 1243-bp fragment for the *phyA-211* mutant. The polymerase chain reaction (PCR)-based markers used to identify the *phyB-9* and *cry1-103* mutants are described by Ward *et al.* (2005). Identification of *bas1-2* and *sob7-1* is described in Turk *et al.* (2005). Photoreceptor mutants were crossed with *bas1-2 sob7-1*, and multiple mutant combinations were isolated in F2 populations.

Due to the use of a large number of higher-order null-mutant combinations in this study, it was not appropriate to use a sibling wild-type Col-0 derived from any one cross as a control. Therefore, a common Col-0 strain was used as a wild-type control in all the experiments for uniformity. To address variation due to environmental growth conditions, all genotypes were grown at the same time under the same growth chamber conditions and seeds harvested for phenotypic analysis.

For generating BAS1:GUS translational fusion lines, the *Bam*HI/*Nco*I fragment from pED10 (described in Turk *et al.* 2003) was cloned into the *Bam*HI/*Nco*1 site of pCAMBIA1305.1 vector (Cambia, Canberra, Australia). This construct was transformed into *bas1-2 sob7-1* double-null plants. Multiple transgenic lines segregating at a 3:1 ratio (hygromycin resistant/sensitive ratio) in the T2 generation were identified as single insertion lines. The entire *SOB7* gene from ∼2.1-Kb upstream of the start codon to the last base before the stop codon was cloned in frame with *uidA* gene in pCAMBIA1305.1 vector. This construct was also transformed into *bas1-2 sob7-1* double-null plants. The segregation ratio was studied to identify multiple single locus T-DNA insertion lines.

### Seed sterilization, plating, and growth conditions

Seeds were surface sterilized by 15 min of agitation in 70% (v/v) ethanol with 0.05% (v/v) TritonX-100 followed by 5 min of agitation in 95% (v/v) ethanol with 0.05% (v/v) Triton X-100 before being air-dried on 90-mm filter paper in a sterile Petri dish. Sterilized seeds were plated on growth media plates containing 1% (w/v) phytagel (Sigma-Aldrich, St. Louis, MO) with 1.5% (w/v) sucrose and 1/2X Linsmaier and Skoog basal media (*Phyto*technology Laboratories; Shawnee Mission, KS). Plates were incubated in darkness at 4° for 4 days. Germination was induced with a red-light treatment at a fluence rate 60-70 µmol m^-2^ sec^-1^ for 16 hr at 25° before being transferred to the appropriate light or dark conditions for a total of 5 days at the same temperature. White light was provided by eight fluorescent tubes (F17T8 17WT; GE Fairfield, CT) and two incandescent tubes (T10 FR25 130V; Satco, Brentwood, NY) in a temperature-controlled growth chamber (Model E-30B; Percival Scientific, Perry, IA).

### Flowering-time analysis

Because transplanting seedlings to soil can cause stresses that alter flowering time, all seeds were directly sown in pots containing a prewatered soil mix (Sunshine Mix4 [Aggregate] LA4; Green Island Distributers Inc., Riverhead, NY). These pots were then incubated in darkness for 4 days at 4° to induce near-uniform germination. Pots were then transferred to growth chambers with white light (200 µmol m^-2^ sec^-1^) set at 21° and 60–70% humidity. After a week of growth, seedlings were thinned to one per pot by clipping using small scissors because removing whole seedlings causes root damage to neighboring seedlings, which in turn can also lead to altered flowering time. We have found that this approach gives the most uniform and repeatable flowering time results for each genotype. Flowering time for both long-day– and short-day–grown plants was calculated by the number of days until the floral stem was 0.5 cm above the basal rosette. In long-day–grown plants, flowering time also was calculated by the total number of primary rosette and cauline leaves present at bolting. This latter approach was not usable with short-day grown plants due to senescence of older leaves during the prolonged growth period for some genotypes.

### Statistical analysis

All statistical results were obtained from at least three independent experiments. Each independent experiment showed the same statistical trend. Results are presented as mean values for the combined data. Error bars represent the mean (SE). A Student’s unpaired two-tailed *t*-test was used to calculate *P*-values that allowed identification of statistically significant differences between two genotypes in a given experiment.

### Histochemical GUS analysis

Plant material was incubated overnight in a GUS staining solution containing 100 mM sodium phosphate, pH 7.0; 10 mM EDTA; 0.5% v/v Triton X-100; 0.5 mM potassium ferri- and ferrocyanide; and 1 mM X-GlucA (Research Product International Corp., Mount Prospect, IL). After staining, plant material was treated with 70% ethanol for 1 hr, followed by 100% ethanol overnight, to remove chlorophyll before photographing. For histological GUS analysis of BAS1 expression in the shoot apex, shoot apices of transgenic plants were dissected with twin blades and GUS stained as described previously. For embedding, tissues were rinsed in 0.1M phosphate buffer (pH 7.0) and embedded in Tissue-Tek OCT (Rankin Biomedical, Holly, MI) compound for sectioning. Embedded tissues were stored at −20° until sectioned. Longitudinal sections were cut using a Leica Cryocut 1800 (Leica Reichert-Jung 1800 Cryostat; Rankin Biomedical, Holly, MI) and image analyzed by Olympus BH-2 Light microscope at the Washington State University Francschi Microscopy and Imaging Center.

### Transcript analysis

Total RNA was isolated, using the RNeasy Plant Kit (QIAGEN, Valencia, CA), from 4-day-old seedlings grown in continuous white light (45 µmol m^-2^ sec^-1^). On-column DNase digestion was performed using the RNase-Free DNase Set (QIAGEN) to eliminate genomic DNA contamination. Total cDNA was synthesized using SuperScriptIII First-Strand Synthesis System (Invitrogen, Carlsbad, CA). *BAS1* transcript was amplified using primers 5′-GCT TAA AAC GTT GAG TAT GAT C-3′ and 5′-TCC TCA TGA TTG GTC AAT CTC-3′. *SOB7* transcript was amplified using primers 5′-CCT GAA AGT CGT AAC AAT GAT C-3′ and 5′-GTT TTC GGA TGA TCA AAT GAG C-3′. *ACTIN2* was used as an internal control in RT-PCR. *ACTIN2* transcript was amplified using primers 5′-GGT CGT ACA ACC GGT ATT GTG CTG G-3′ and 5′-CTG TGA ACG ATT CCT GGA CCT GCC-3′. The linear range of amplification for each gene transcript was determined by comparing samples obtained using different numbers of cycles. Lack of genomic and foreign DNA contamination was ascertained by using all RNA samples and water as a template in a PCR reaction.

## Results

### Removal of *BAS1* and *SOB7* rescues the low germination rate of a *phyA phyB* double-null mutant

Flowering-time analysis required seeds to be directly sown on the soil. Because the number of days to flowering were calculated based on the day of planting, it was important to know whether the genotypes in this study conferred any germination phenotypes. For this purpose, a seed germination study was conducted in the lab.

In both light and dark conditions, the *bas1-2* and *sob7-1* single and *bas1-2 sob7-1* double null did not show a significant difference in germination rates when compared with the wild-type Col-0 (in white light, *P* = 0.18, 0.64, and 0.27 respectively; in dark, *P* = 0.45, 0.33, and 0.29, respectively). The *phyA-211 phyB-9* double mutant conferred a lower germination percentage rate, both in darkness and after a prolonged white light treatment (Table 1). In contrast, the *phyA-211 phyB-9 bas1-2* and *phyA-211 phyB-9 sob7-1* triple-null mutants displayed significantly greater germination rates than *phyA-211 phyB-9* in both white light (*P* = 0.0025 and 0.0018, respectively) and dark treatments (*P* = 0.0001 and 0.0002, respectively). The *phyA-211 phyB-9 bas1-2 sob7-1* quadruple-null displayed the greatest germination rate, which was similar to the wild type in both dark and light treatments (*P* = 0.79 and 0.52 respectively). These results suggest that *BAS1* and *SOB7* are acting downstream and/or in parallel with phyA and phyB for modulating seed germination.

**Table 1:** Percentage of seed germination in white light or darkness

	**Wild type**	***bas1-2***	***sob7-1***	***bas1-2 sob7-1***
Shifted to dark	95.6 (1.1)	97.2 (1.8)	92.8 (2.5)	97.2 (1.0)
White light	97.7 (1.1)	91.1 (4.0)	96.7 (1.9)	96.7 (3.3)
	***phyA***	***phyA bas1-2***	***phyA sob7-1***	***phyA bas1-2 sob7-1***
Shifted to dark	86.7 (5.7)	94.9 (1.9)	95.5 (1.9)	96.7 (1.2)
White light	95.5 (2.2)	94.4 (4.0)	94.4 (4.0)	100 (0)
	***phyB***	***phyB bas1-2***	***phyB sob7-1***	***phyB bas1-2 sob7-1***
Shifted to dark	95.5 (3.3)	94.4 (1.8)	93.3 (2.6)	95.5 (2.2)
White light	95.5 (1.1)	91.1 (2.9)	96.7 (1.9)	90.0 (6.9)
	***phyA phyB***	***phyA phyB bas1-2***	***phyA phyB sob7-1***	***phyA phyB bas1-2 sob7-1***
Shifted to dark	20.4 (5.8)	83.9 (3.7)	87.2 (4.7)	95.0 (1.7)
White light	35.6 (6.7)	85.6 (2.9)	87.8 (2.2)	98.9 (1.1)

Imbibed seeds were incubated at 4° in the dark for 4 days before being treated with white light for 6 days (three replicates) or with white light for 1 day followed by 5 days in darkness (six replicates) at 25°. Values in brackets represent the SE of the mean

### *BAS1* and *SOB7* show genetic interactions with photoreceptors for flowering time

**Figure 1:**
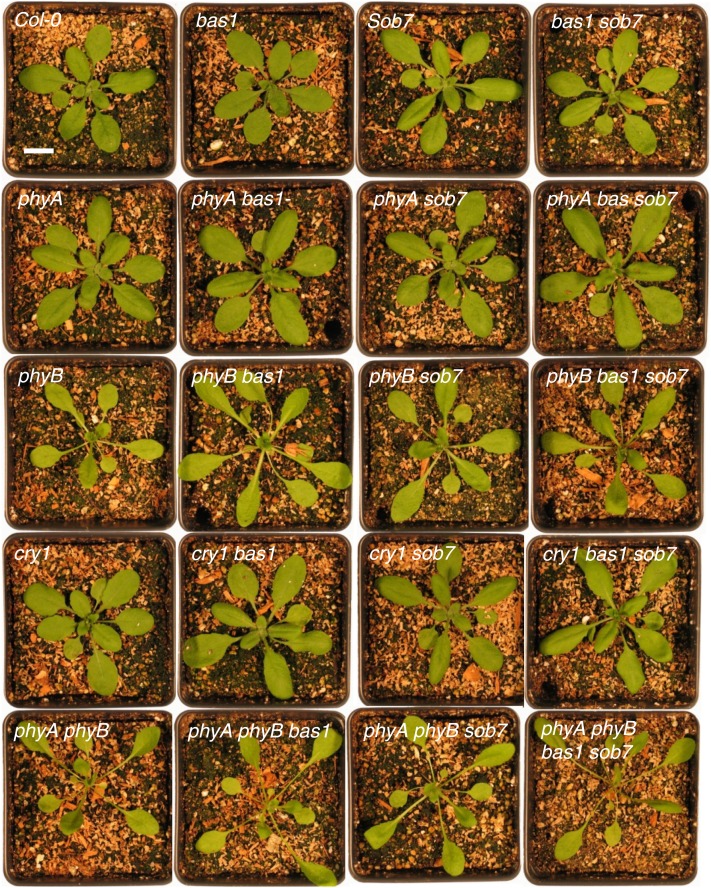
Adult phenotype of the genotypes used in this study. Wild-type and single-, double-, and multiple-mutant genotypes were grown in long-day conditions for 3 weeks before being photographed. Scale bar = 1 cm.

To further examine genetic interactions between the BR-inactivating enzymes and photomorphogenic photoreceptors, we measured flowering time in multiple null mutant combinations of *bas1-2* and *sob7-1* with *phyA-211*, *phyB-9*, *cry1-103*, and *phyA-211 phyB-9* grown in both long- (16-hr light:8-hr darkness) and short-day (8-hr light:16-hr darkness) conditions. Adult plant phenotypes of the various genotypes grown for 3 weeks under long-day conditions are shown in Figure 1.

**Figure 2:**
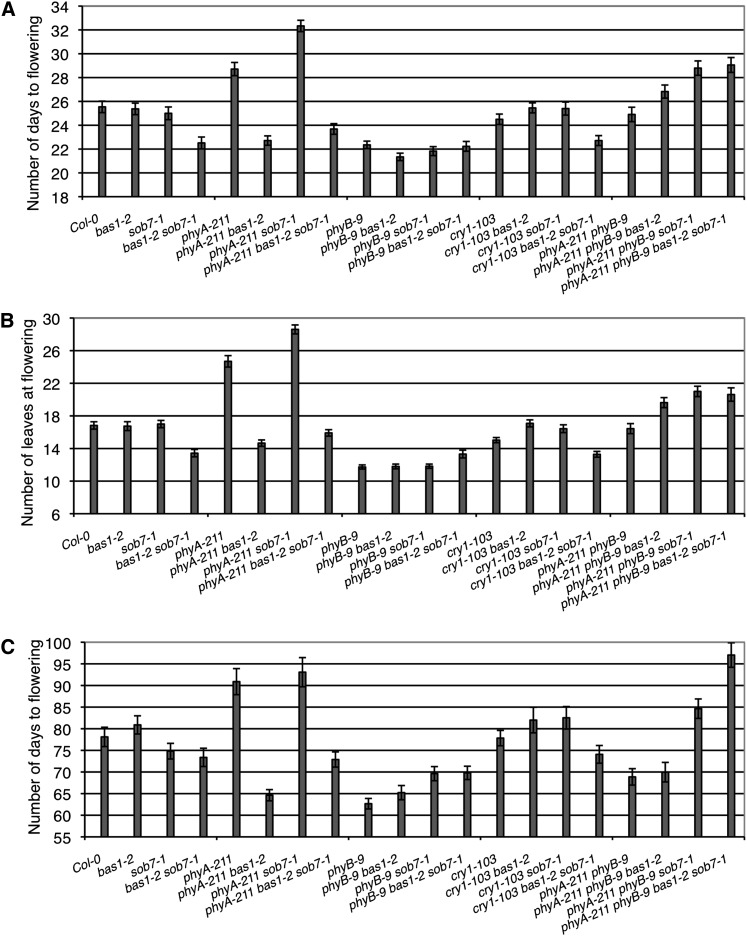
Genetic interactions between *BAS1*, *SOB7*, *PHYA*, and *PHYB* to control flowering time. The *bas1-2* but not the *sob7-1* mutant suppresses the late-flowering phenotype of *phyA-211* mutants in both long day (A and B) and short days (C). *phyB-9 bas1-2 sob7-1* triple- and *phyA-211 phyB-9 bas1-2 sob7-1* quadruple-null did not flower earlier than the *phyB-9* single- and *phyA-211 phyB-9* double null, respectively, suggesting that the *bas1-2 sob7-1* early-flowering phenotype requires functional phyB (A and B). Three replications with 10 plants per replication were used for flowering analysis. Error bars represent SE.

Flowering time analysis demonstrated complex genetic interactions between these various genes for determination of flowering time. These genetic interactions were maintained in both long- and short-day growth conditions (Figure 2). The genetic state of *CRY1* did not have any impact on floral induction or the early-flowering phenotype conferred by the loss of *BAS1* and *SOB7*. In contrast, the *bas1-2* mutation suppressed the *phyA-211* late-flowering phenotype in both long and short days. However, the *sob7-1* mutation had the opposite effect on flowering time in combination with *phyA-211*. In addition, *phyB-9 bas1-2 sob7-1* triple-null and *phyA-211 phyB-9 bas1-2 sob7-1* quadruple-null plants did not flower earlier than the *phyB-9* single-null and *phyA-211 phyB-9* double-null controls, respectively, suggesting that the *bas1-2 sob7-1* early-flowering phenotype requires functional phyB.

### BAS1 and SOB7 have distinct and overlapping expression patterns

To gain further insight into the function of BAS1 and SOB7, we generated translational GUS fusion lines of BAS1 and SOB7 under the control of their native promoters. These *BAS1*:BAS1-GUS and *SOB7*:SOB7-GUS transgenes were expressed in the *bas1-2 sob7-1* double-null mutant background. Single-locus insertion lines were isolated and characterized via molecular and genetic analyses (Supporting Information, Figure S1). Transformation into the *bas1-2 sob7-1*–null background provided transgene stability by reducing the chance of cosuppression from the endogenous transcript. In addition, it also provided an opportunity for functional analysis of the transgenic lines by complementation analysis of the double-null hypocotyl elongation phenotype.

The hypocotyl elongation phenotype of *bas1-2 sob7-1* was rescued by *BAS1*:BAS1-GUS translational fusions in accordance with the expression level from the transgene (Figure S1, A and B). However, all *SOB7*:SOB7-GUS lines except one have a similar hypocotyl-elongation phenotype as the double mutant regardless of the level of gene expression (Figure S1, C and D). This observation can be explained on the basis that *BAS1* and *SOB7* are not completely redundant for hypocotyl growth (Turk *et al.* 2005). Because the p*SOB7*:SOB7-GUS was transformed into *bas1-2 sob7-1* double-null background, the p*SOB7*:SOB7-GUS transgenic lines shown in Figure S1C are still lacking BAS1 activity. Therefore, the restoration of *SOB7* to its wild-type expression level by the transgenic construct does not rescue the *bas1-2 sob7-1* hypocotyl phenotype completely. Only when the expression from the transgenic p*SOB7*:SOB7-GUS construct is significantly greater than the wild-type level, as is the case in line #3.3, does it significantly shorten the hypocotyl of the *bas1-2 sob7-1* double-null line.

**Figure 3:**
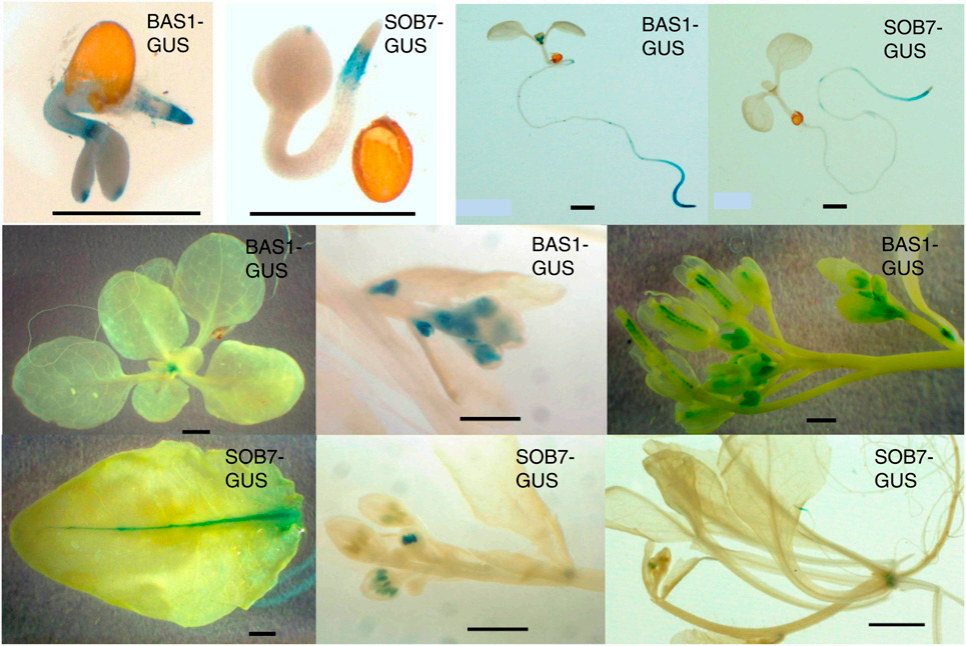
Expression patterns of BAS1 and SOB7. GUS staining patterns of the seedlings or tissues from *BAS1*:BAS1-GUS and *SOB7*:SOB7-GUS transgenic plants at various developmental stages. Scale bar = 1.0 mm.

Histochemical GUS analysis was performed on a set of representative lines. In white light–grown seedlings, BAS1 expression was observed in the shoot apex and root tip (Figure 3). In contrast, SOB7 expression was seen only in the root elongation zone (Figure 3). In juvenile and adult plants, BAS1 expression was present in the shoot apex before flowering, and in the flowers and developing embryos after flowering (Figure 3). SOB7 expression was present in the transition zone between the root and the shoot, as well as in developing anthers, the vasculature of rosette leaves and hydathodes of cauline leaves (Figure 3). Overall, BAS1 and SOB7 had some overlapping but mostly distinct expression patterns.

**Figure 4:**
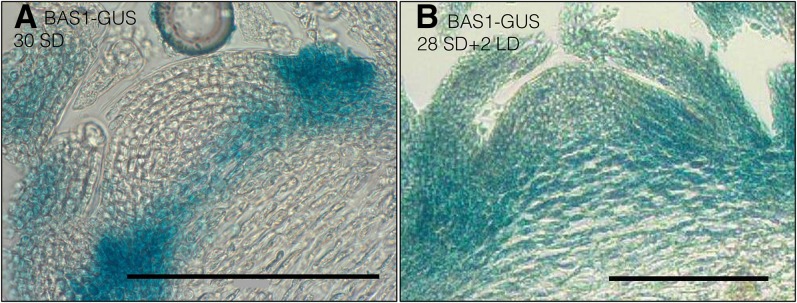
Histochemical GUS analysis of BAS1 expression in shoot apex during transition to flowering. Longitudinal section through the shoot apex of 4-week-old transgenic plant expressing *BAS1*: BAS1-GUS grown in short day conditions (A). Longitudinal section through the shoot apex of 4-week-old transgenic plants expressing *BAS1*: BAS1-GUS after shifting to long day conditions for 2 days (B). Scale bar = 0.1 mm.

The early-flowering phenotype of *bas1-2 sob7-1* and expression of BAS1-GUS in the shoot apex suggests that BAS1 is involved in regulating BR levels in the shoot apical meristem, which may in turn affect the vegetative to floral phase transition. To test this hypothesis transgenic plants carrying the p*BAS1*:BAS1-GUS construct were grown in short-days for 4 weeks. After 4 weeks, half of the plants were shifted to long days whereas the remaining half stayed in the short days. After two additional days, tissue from plants that stayed in the short days for the entire time and plants that were shifted to the long days were collected together. As a result, at the time of collection, all plant tissues were the same age. These harvested tissues were immediately used for histological analysis. Histochemical GUS analysis demonstrated a change in the expression pattern of BAS1 during this phase transition from short-day to long-day growth conditions (Figure 4). In short-day-grown plants (Figure 4A), expression was only visible at the base of the shoot apex, whereas after floral induction via transfer to long-day growth conditions BAS1-GUS expression was present throughout the shoot apex (Figure 4B). This observation, that the change in BAS1 expression pattern is correlated with floral induction suggests a role for BAS1 in flowering.

### BAS1 expression in the shoot apex in red light is dependent on the presence of functional phyB

**Figure 5:**
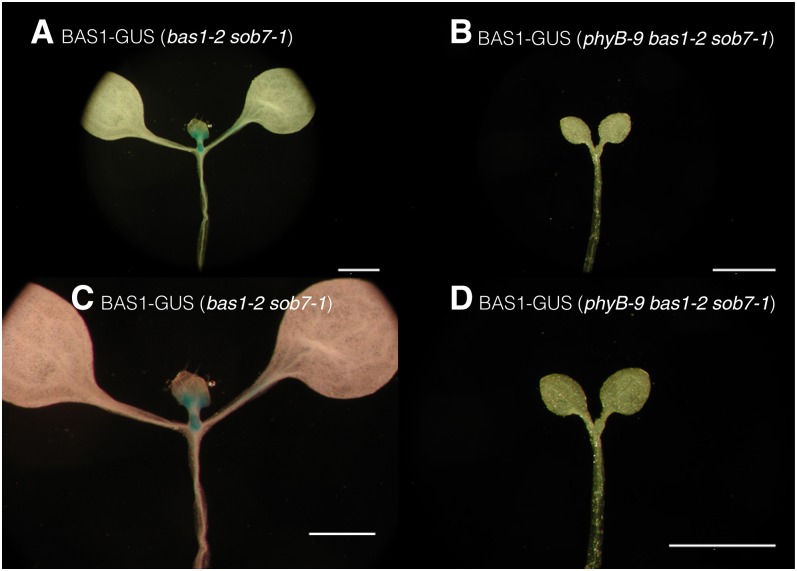
BAS1 expression in the shoot apex in red light is dependent on the presence of functional phyB. Transgenic seedlings carrying identical transgenic event of p*BAS1*: BAS1-GUS construct in the *bas1-2 sob7-1* background (A and C) and *phyB-9 bas1-2 sob7-1* triple-null background (B and D). Seedlings were grown in 45 µmol m^-2^ sec^-1^ of red light for five days before histochemical GUS analysis. Scale bar = 1 mm.

Based on the observation that BAS1-GUS expression is present in the shoot apex and that the early-flowering phenotype of *bas1-2 sob7-1* is dependent on the presence of functional phyB, we hypothesized that phyB signaling is regulating BAS1 expression in the shoot apex which in turn affects flowering time. To test this hypothesis, multiple p*BAS1*:BAS1-GUS transgenic lines in the *bas1-2 sob7-1* background were crossed with the *phyB-9 bas1-2 sob7-1* triple null to isolate BAS1-GUS translational fusions in both the wild-type *PHYB* and *phyB-9*–mutant genetic backgrounds. BAS1-GUS fusions in the *bas1-2 sob7-1*, and *phyB-9 bas1-2 sob7-1* background were grown in continuous red light for 5 days. BAS1-GUS expression was examined in the shoot apex by histochemical GUS analysis in the genotypes. Using this approach, we observed that the BAS1-GUS expression in the shoot apex was much more prominent in the wild-type *PHYB* background than the *phyB-9* background (Figure 5). This observation suggests that BAS1 expression at the shoot apex is modulated by the presence of functional phyB. It also suggests a possible cause for the dependence of the early-flowering phenotype of *bas1-2 sob7-1* on functional phyB.

## Discussion

As previously reported, the *phyA-211 phyB-9* double mutant displayed a lower germination rate than the wild type (Poppe and Schafer 1997), which was rescued by the removal of *BAS1* and *SOB7* (Table 1). This implies that both are acting either downstream of or in parallel with *PHYA* and *PHYB* to modulate seed germination presumably by changing levels of active BRs. In comparison with germinating seeds, flowering-time phenotypes in adult plants cannot be interpreted solely based on the changing of overall BR levels.

Flowering-time analysis of mutants blocked in BR biosynthesis (*e.g.*, *det2*, *dwf4*, and *cpd*) or BR perception (*bri1*) suggests a positive role for BRs in floral induction (Chory *et al.* 1991; Li and Chory 1997; Azpiroz *et al.* 1998; Domagalska *et al.* 2007). However, transgenic *Arabidopsis* plants having constitutive overexpression of *DWF4*, with higher levels of active BRs and increased organ size, do not display early flowering, suggesting that the interplay between BRs and flowering is not simply a matter of altering whole-plant hormone levels (Choe *et al.* 2001). The *bas1-2 sob7-1* double mutant contains higher BR levels and flowers earlier than the wild type, demonstrating a role for these BR-inactivating enzymes in floral induction (Turk *et al.* 2005).

Our results show that the *bas1-2* mutation suppresses the late-flowering phenotype of *phyA-211*, whereas the *sob7-1* mutation does not (Figure 2), suggesting that these two BR-inactivating genes can have distinct roles in plant development. Unlike BAS1, SOB7 is not expressed in the apical meristem in seedlings as well as adult plants (Figure 3). Because the transition to flowering occurs at the shoot apical meristem, these differences in expression can, in part, explain the distinct genetic interactions that *bas1-2* and *sob7-1* have with *phyA-211*.

In addition, we also observed that in short-day grown-plants that have not been induced to flower, BAS1 expression is confined mainly to the basal region of shoot apex (Figure 4A). In contrast, after floral induction, BAS1 expression is seen throughout the shoot apex (Figure 4B). The expression of BAS1 at the base of the shoot apex may be involved in excluding BRs from the shoot apical meristem to prevent an early transition to flowering. Interestingly, a GA catabolic enzyme encoding gene, *OsGA2ox1*, implicated in vegetative to floral phase transition in rice, also shows a similar expression pattern (Sakamoto *et al.* 2001).

The *bas1-2 sob7-1* double mutant also displayed a genetic interaction with *phyB-9* with regard to flowering time (Figure 2, A and B). The observation, that both the *phyB-9* single-null and *phyB-9 bas1-2 sob7-1* triple-null flower at the same time, suggest that the early-flowering phenotype of *bas1-2 sob7-1* requires functional phyB. Loss of BAS1 expression in the *phyB-9* background in red light (Figure 5, B and D) also suggests a possible molecular basis for the genetic interaction of *BAS1* and *SOB7* with *PHYB* in regard to floral induction. Another possible explanation is that BAS1 expression in the shoot apex is dependent on certain morphological and physiological attributes that are lacking in the *phyB-9* mutant background. In this case, the requirement of functional phyB would be indirect rather than direct. Examples of two such morphological attributes that are altered in *phyB-9* seedlings grown in red light are smaller cotyledons and petioles when compared to the wild type (Neff and Van Volkenburgh 1994; Neff and Chory 1998), a phenotype that is obvious in the Figure 5. In contrast to *PHYA* and *PHYB*, the genetic state of *CRY1* had no impact on the early-flowering phenotype conferred by the *bas1-2 sob7-1* double null. These results demonstrate that higher-order null-mutant analysis can be used to differentiate the roles of different photoreceptors in BAS1- and SOB7-mediated development.

To fully understand the role of BAS1 in flowering, it will be necessary to study the changes in its expression pattern in relation to cellular or tissue-specific BR levels in the shoot apex, during floral induction, in both the wild-type and *phyB-9* mutant backgrounds. However, we currently lack the technology to accurately measure BR levels in small tissues and organs such as the shoot apical meristem. Such an advancement, which could include a DR5-like reporter system for BR levels (Ulmasov *et al.* 1997), would further help in understanding the overall role of BR catabolism in plant growth and development.

**Figure 6:**
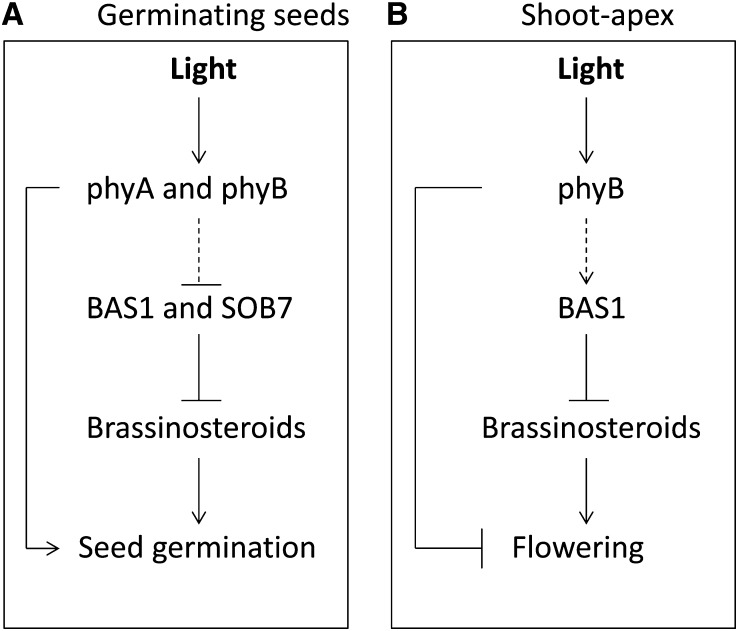
Model based on the interpretation of the genetic interactions between photomorphogenic photoreceptors and *BAS1* and *SOB7*. (A) In the seed, *BAS1* and *SOB7* act downstream and/or in parallel of phyA and phyB to promote germination. (B) In the shoot-apex, phyB modulate BAS1 expression to inhibit phase transition. The dashed arrows indicate the suggested interactions based on this study.

A simplified model describing the suggested roles of BAS1 and SOB7 in seed germination and floral induction is shown in Figure 6, A and B, respectively. BRs are known to have a positive effect on seed germination (Steber and McCourt 2001). Therefore, germination may include an increase in BR levels via the regulation of BR catabolism. phyA and phyB also affect germination (Poppe and Schafer 1997). These two processes are either acting independently, interdependently or both. As shown in the Figure 6A, *BAS1* and *SOB7* may act downstream of and/or in parallel to phyA and phyB to promote germination.

In the shoot apex, the transition to flowering includes changes in BAS1’s expression pattern (Figure 4), suggesting that there might be a cause and/or effect relationship between the two events. phyB plays an inhibitory role in flowering (Goto *et al.* 1991; Whitelam and Smith 1991; Halliday *et al.* 1994). phyB is required for the early flowering phenotype conferred by the *bas1-2 sob7-1* double mutant (Figure 2) and alters the expression pattern of BAS1 (Figure 5). Therefore, it is possible that phyB modulates BAS1 expression to inhibit phase transition in the shoot apex. Figure 6B depicts a possible mechanism for the inhibition of flowering at the shoot apex with regard to genetic interactions between phyB and BAS1. In addition to *BAS1* and *SOB7*, at least five more genes (*BEN1*, *UGT73C5*, *UGT73C6*, *ATST4a*, and *BIA1*) have been suggested to play a role in BR catabolism (Poppenberger *et al.* 2005; Marsolais *et al.* 2007; Yuan *et al.* 2007; Husar *et al.* 2011; Roh *et al.* 2012). Delineating the overall role of BRs and BR catabolism in plant physiology and development is likely to include a similar molecular genetic approach as described in this study.

## Supplementary Material

Supporting Information
