# Supplementary material for: Genetic Interactions Between Brassinosteroid-Inactivating P450s and Photomorphogenic Photoreceptors in Arabidopsis thaliana
Source: G3 (Bethesda). 2012 Dec 1;2(12):1585–93. doi: 10.1534/g3.112.004580 (PMC3516480; doi:10.1534/g3.112.004580)
Supplement: Supporting Information [file supp_2_12_1585__index.html]

Supporting Information 

# Genetic Interactions Between Brassinosteroid-Inactivating P450s and Photomorphogenic Photoreceptors in *Arabidopsis thaliana*

## Supporting Information for Sandhu, Hagely, and Neff, 2012

**Files in this Data Supplement:**

- Supporting Information - Figure S1 and File S1 (PDF, 690 KB)
- Figure S1 - Genetic and molecular analysis of *BAS1*:BAS1-GUS and *SOB7*:SOB7-GUS lines (PDF, 687 KB)
- File S1 - BAS1-GUS hypocotyl data, SOB7-GUS hypocotyl data, flowering data in long-day, flowering data in short-day, and genotype data (.zip, 117 KB)
