# Supplementary material for: Genetic Interactions Between Brassinosteroid-Inactivating P450s and Photomorphogenic Photoreceptors in Arabidopsis thaliana
Source: G3 (Bethesda). 2012 Dec 1;2(12):1585–93. doi: 10.1534/g3.112.004580 (PMC3516480; doi:10.1534/g3.112.004580)
Supplement: Supporting Information [file supp_2.12.1585_004580SI.pdf]

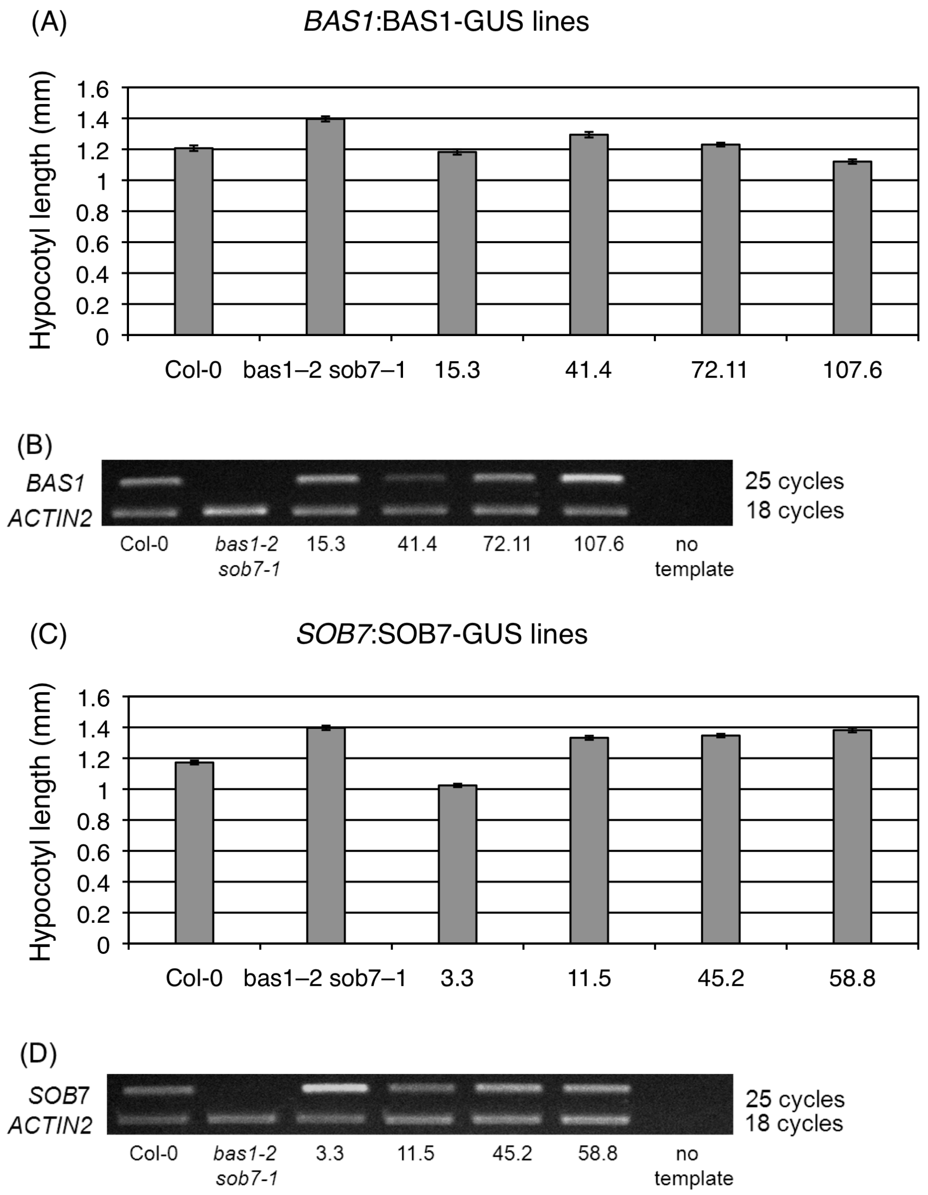

Figure S1 Genetic and molecular analysis of *BAS1*:BAS1-GUS and *SOB7*:SOB7-GUS lines. Genetic and molecular analysis of transgenic plants expressing *BAS1*:BAS1-GUS (A and B) and *SOB7*:SOB7-GUS (C and D) in *bas1-2 sob7-1* background. Seedlings were grown in 45 µmol m^{-2} sec^{-1} of white light for four days before being digitized and measured. For transcript analysis total RNA was isolated from four-day-old seedlings grown in similar conditions as used for hypocotyl growth analysis.

File S1 Supporting Data

A compressed folder is available at http://www.g3journal.org/lookup/suppl/doi:10.1534/g3.112.004580/-/DC1

Files include:
BAS1-GUS hypocotyl data
SOB7-GUS hypocotyl data
Flowering data in long-day
Flowering data in short-day
Genotype data
