# Supplementary material for: Genetic Interactions Between Brassinosteroid-Inactivating P450s and Photomorphogenic Photoreceptors in Arabidopsis thaliana
Source: G3 (Bethesda). 2012 Dec 1;2(12):1585–93. doi: 10.1534/g3.112.004580 (PMC3516480; doi:10.1534/g3.112.004580)
Supplement: Supporting Information [file supp_2.12.1585_FigureS1.pdf]

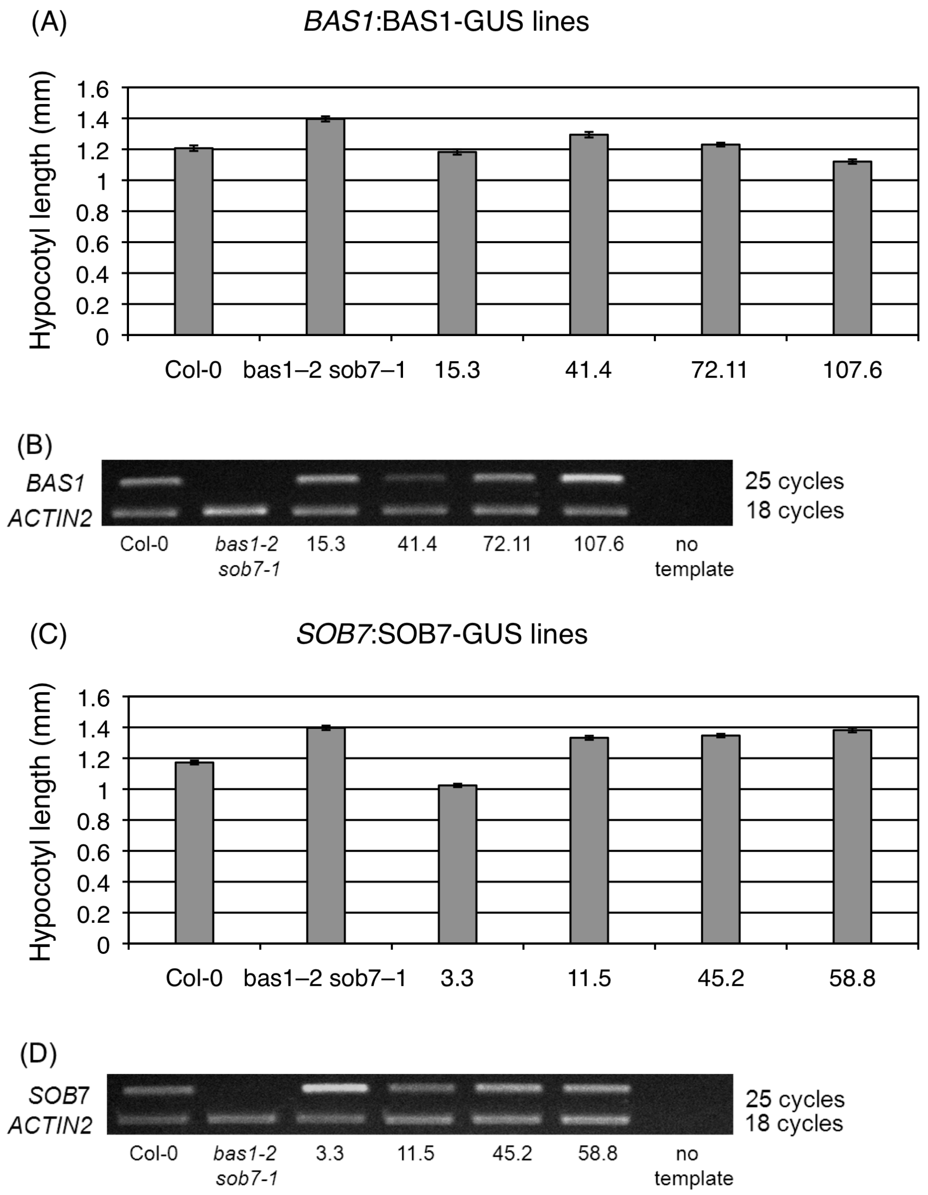

Figure S1 Genetic and molecular analysis of *BAS1*:BAS1-GUS and *SOB7*:SOB7-GUS lines. Genetic and molecular analysis of transgenic plants expressing *BAS1*:BAS1-GUS (A and B) and *SOB7*:SOB7-GUS (C and D) in *bas1-2 sob7-1* background. Seedlings were grown in 45 µmol m^{-2} sec^{-1} of white light for four days before being digitized and measured. For transcript analysis total RNA was isolated from four-day-old seedlings grown in similar conditions as used for hypocotyl growth analysis.
